# 3D nanopetrography and chemical imaging of datable zircons by synchrotron multimodal X-ray tomography

**DOI:** 10.1038/s41598-018-22891-9

**Published:** 2018-03-16

**Authors:** J.-P. Suuronen, M. Sayab

**Affiliations:** 1 ESRF – The European Synchrotron, CS40220, 38043 Grenoble Cedex 9, France; 2 Geological Survey of Finland, P.O. Box 96, FI-02151 Espoo, Finland

## Abstract

Zircon is the most widely used mineral in petrochronology and provides key information about magmatic and crustal differentiation history of plutonic rocks, transport paths of clastic material ‘from source to sink’ and significantly contributes in the reconstruction of enigmatic planetary-scale tectonic episodes since the Archaean. However, detailed textural analysis of this accessory mineral has always been hampered by two-dimensional (2D) analytical limitations. With the advancements in X-ray nanotomography technology, it is now possible to non-destructively, yet digitally, cut, visualize, compare and quantify internal textures within zircons, their growth and zoning patterns and chemical distribution of trace elements in three dimensions (3D). We present a novel multimodal approach of using a synchrotron radiation nanobeam to perform 3D nanopetrography of < 100 µm zircons at ~100 nm resolution, demonstrating the capabilities of the technique by analysis of Paleoproterozoic zircons from the Central Finland Granitoid Complex. The integrated X-ray absorption, diffraction and fluorescence tomography revealed sector and oscillatory zoning patterns in 3D as well as differences in zoning pattern between trace elements, in addition to lattice parameters and inclusion composition within zircons. The multimodal synchrotron nanotomography elucidates the 3D nanopetrography and trace element composition of submillimeter-sized zircons in unprecedented detail.

## Introduction

Zircon has been the best choice for decades to date and correlate geological events of the Earth’s history as it preserves long-lived isotope systems^1,2^. The mineral offers a range of isotopic systematics and trace element distribution to investigate petrochronological information, such as magmatic and crustal differentiation history of granitic plutons and their petrogenesis^3^. In many studies, the isotopic signatures of zircon have made promises to detect provenance of detrital sediments and to trace supercontinent reconstructions from Nuna to Pangaea^4–6^. Uranium and thorium are readily incorporated into zircon as trace elements, whereas lead isotopes (^206^Pb, ^207^Pb and ^208^Pb) in the crystal are produced by radioactive decay from ^238^U, ^235^U and ^232^Th, allowing the age of the crystal to be determined by isotopic dating. U-Pb dating, however, is not necessarily a straightforward process, as the chronological history of the zircon crystal can manifest itself as recrystallization leading to preferential expulsion of Pb from the zircon lattice and influence the age determination^2^. This is a potential source of ambiguity in age determination based on whole zircon crystals, and spatially resolved dating techniques such as laser ablation inductively coupled plasma mass spectrometry (LA-ICP-MS) and secondary ion mass spectrometry (SIMS) are commonly used to ensure that isotopic measurements are taken only within homogeneous age domains in the crystal^7–10^. While the sensitive high-resolution ion microprobe (SHRIMP)^11–13^ is capable of producing spot sizes down to a few micrometres, and the nanoSIMS instrument^14^ can even reach sub-micrometre resolution, these methods are generally used for spot analysis or scanning small regions on the surface of a sample to relate the age and chemical information to specific zones of the zircon structure. The need to couple them with imaging techniques, such as backscattered and scanning electron imaging (BSE, SE), electron backscatter diffraction (EBSD) and Raman spectroscopy mapping, has long been recognized^15–19^.

A conspicuous feature commonly visualized in zircon with high-resolution microscopic techniques is growth zoning, or variations of trace element concentration showing up as grayscale variation in CL and BSE images. In addition to aiding in selecting unaltered zircons (or domains within one zircon) for U-Pb dating^20^, the internal texture of zircons is representative of the petrochronological history such as (re)crystallization, metamorphic events and metamictization^21,22^. Although capable of visualizing the internal textures of zircon at high resolution, both CL and BSE imaging share the same unavoidable drawbacks. As essentially 2D techniques, they can only image the surface of the section cut through the grain, and for 3D imaging require serial polishing and intermittent imaging of the grain: the polished-off part is lost to any further analysis. LA-ICP-MS provides fine chemical details across the 2D polished surface, but the technique by no means provides additional textural information of what lies underneath the surface. In particular, conventional laser-ablation spot size is typically 20–40 µm in diameter. With a pit depth of 30–50 µm, ICP-MS analyses have higher chances to yield analyses of mixed domains, especially in the case of small multiple zircon growths.

In this study, we present a 3D imaging approach coupled with non-invasive 2D virtual cross-sections to analyse the nanopetrography, texture, chemical composition, zoning and inclusion mineralogy of zircon with ~100 nm resolution using synchrotron radiation on the newly developed nanofocus beamline ID16B^23^ of the European Synchrotron (http://www.esrf.fr/UsersAndScience/Experiments/XNP/ID16B). Combining phase-contrast X-ray nanotomography^24,25^ with X-ray absorption, fluorescence, and diffraction allows a more thorough view of the zircon internal structure. Key questions can be resolved before any spot (e.g., laser ablation) analysis, for example: What lies in the 3^rd^ dimension of the zircon? How is the zoning pattern distributed in 3D? How many inclusions are there in the grain, and what are their sizes and composition? How are different age domains (e.g. core and rim overgrowths) spatially distributed? What is the density of fractures and how are the fractures linked in the 3^rd^ dimension? Where to place the spot to avoid fractures and inclusions? The technique presented here can answer these questions by mapping the datable domains in 3D down to ~100 nm. Moreover, zircons from extraterrestrial samples or those preserving Hadean ages can first be scanned at high spatial resolution for archival purposes before being exposed to non-destructive procedures.

For this study, we chose three paleoproterozoic igneous zircons from the Central Finland Granitoid Complex (CFGC), to image and further analyse the different trace elements in their oscillatory and sector zoning patterns (as described by Corfu *et al*.^22^), fracture patterns, strain of the zircon lattice, and mineralogy of inclusions with fluorescence and diffraction measurements. By identifying the same features in 3D nanotomography reconstructions, this chemical and crystallographic information can be extrapolated to the entire volume of the zircon crystal. We envisage that there is a good potential that the method can equally be applied to detrital zircons, where 20 or more representative zircons from a rock sample can be scanned at relatively low resolution (~400 to 500 nm), before mounting them along with other unscanned zircons extracted from the same sample. After mounting and polishing, the 2D crystal surface can be matched with the 3D visuals of the scanned zircons and the spot location (e.g., laser ablation) can be placed without any doubts^25^. Once analysed after ablation, the method lends itself to comparing errors in the age and/or geochemical analysis between the scanned and unscanned zircons.

## Sample description

The three zircons examined are from populations that have already been dated (U-Pb, and O isotopes) with the Cameca IMS 1280 ion microprobe of the NordSIMS facility, Swedish Museum of Natural History^26^. The samples represent part of the Central Finland Granitoid Complex (CFGC), which is predominantly composed of intermediate to felsic granodiorites and granites surrounded by supracrustal belts in central Finland. Two zircons were selected from sample A1973 (zircons A1973a, and A1973b), and one from A1971. Both granodiorite samples are deformed and foliated, and classified as I-type granitoids. The zircon U-Pb ages are 1885 ± 3 Ma for A1971 and 1887 ± 4 Ma for A1973^26^. The aim of this work is to demonstrate the non-destructive nature and high resolution multimodal 3D visualization and chemical imaging capabilities of the nanofocus beamline that can be applied to any micrometre sized datable mineral, like zircon: for the detailed isotopic (U-Pb, O, Lu-Hf) and geochemical evolution of these two (A1971 and A1973) and other related samples the reader is directed to the recent article^26^.

## Full-field nanotomography imaging

As a first step of analysis, the zircons were imaged with the X-ray phase-contrast tomography technique called holotomography^24^. In short, a holotomography scan consists of acquiring four sets of radiographs of the sample, rotating the sample 360° during one set, and changing the X-ray propagation distance between the sample and detector between rotations. After alignment of the four sets of radiographs, calculation of phase maps, and tomographic reconstruction of the phase maps, this technique yields a 3D voxel volume of the sample similar to conventional absorption microtomography^27–29^ (µCT), with the difference that the voxel size is smaller (100 nm in this work), and the voxel values reflect the X-ray phase change instead of attenuation coefficient. The differences between attenuation and phase contrast are outlined e.g. by Fusseis *et al*.^30^.

**Figure 1:**
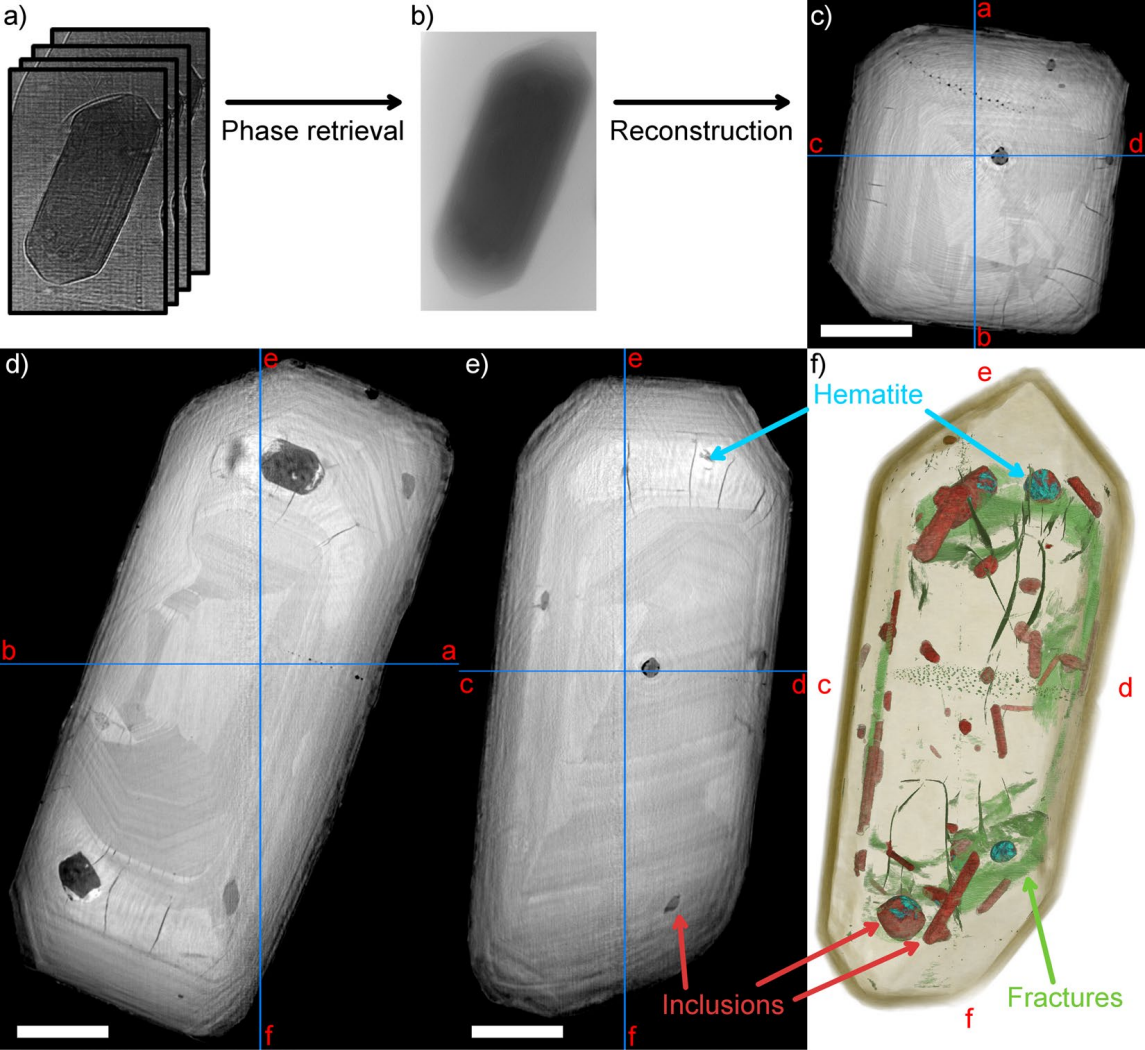
The principle of propagation-based phase contrast tomography and results of zircon 1973A. (**a**) Untreated projection images. The scan consists of acquiring 4 tomographic scans with varying sample-to-detector distance. (**b**) One phase map, i.e. projection image which is the result of passing the four images with different propagation distance through a phase retrieval algorithm. Each pixel in the image represents the phase change of the X-rays traversing through the sample. (**c–e**) Axial (x-y), and two longitudinal (x-z and y-z) slices of the tomographic reconstruction computed based on the phase maps. The blue lines indicate the positions of the orthogonal slices in each. Scale bars are 20 µm. (**f**) 3D rendering of the whole volume (i.e. projection of the 3D structure to the image plane), showing the zircon matrix in brown, porosities and cracks within the zircon in green, inclusions in red, and the dense Fe-rich phase (hematite, cf. Figs 2 and 5) inside the inclusions in blue. A video showing the zircon from different directions is included as video S1 in the supplementary material.

Figure 1 shows the data processing workflow of holotomography, and the obtained 3D structure of zircon A1973a. By viewing cross-sections along the three cardinal axes (Fig. 1c–e), following details can be observed:

- The relatively complex zoning pattern, where oscillatory zoned areas are interrupted by regions that are weakly zoned or with a homogenous appearance.
- A number of fractures in the crystal, cutting, especially, across the homogenous (little or no oscillatory zoning) regions.
- Trails of several aligned small, dark inclusions of 1–5 µm diameter cutting across the middle of the crystal.
- Several inclusions of varying grayvalue and shape, mostly of lower density than the zircon, but with a very bright phase present inside some of the larger inclusions.

The 3D nanopetrography of the inclusions and fractures in the zircon can be visualized and quantified by segmenting the dataset into different phases and using 3D rendering techniques (Fig. 1f, video in supplementary material). A well-defined euhedral shape of the zircon can be seen outlining the crystal geometry, and transparent rendering of the outline yields the internal nanotextures including distribution of fractures, inclusions and pores. Inclusions (in red in Fig. 1f) can be broadly divided into 2 categories: elongated, narrow inclusions with prismatic shape corresponding to those with intermediate grayvalue, and shorter roundish inclusions with a relatively low grayvalue, but containing the majority of the bright phase. Mineralogy of these inclusions is discussed in the X-ray diffraction section below.

Zircons A1971 and A1973b exhibit predominantly similar features as A1973a. Zircon A1971 contains a distinct dark zone, approximately 2 µm thick, surrounding the center of the zircon, which has approximately the same euhedral shape as the whole zircon. Zircon A1973b is subhedral, and exhibits a mostly oscillatory zoning pattern weakly visible in the phase contrast images. The majority of inclusions in A1973b are localized at the center of the crystal, whereas most inclusions in A1971 and A1973a were found to be at the rim regions. A higher-resolution holotomography centered on one of these inclusions with 25 nm voxel size enables identifying at least 5 different phases within the inclusion. The interface between two of these phases appears to form a concave meniscus, suggesting the denser of the two phases might be liquid.

## X-ray absorption and fluorescence Imaging

While mapping the zircon internal structure in 3D, holotomography does not provide any chemical information as to which trace elements are responsible for producing the zoning pattern, or to the exact composition of inclusions. On selected virtual 2D slices through the zircon, the trace element distribution can be obtained from X-ray fluorescence tomography (XRF-CT, see e.g. Wildenschild and Sheppard^31^ for an overview). A detailed description of the data treatment is left to the analytical methods section, but in short, an XRF-CT scan is performed with zircon in the focus of the X-ray beam, so that only a narrow strip of approximately 50 nm × 50 nm through the zircon is illuminated at a time. Scanning the sample across the beam and rotating between line scans, a sinogram is obtained in the pencil beam CT geometry. During the scan, three types of information can be measured:

- The spatial distribution of different elements can be measured by recording the element-specific characteristic X-rays emitted by the sample with an energy sensitive detector
- A pure attenuation image (without any phase effects) of the sample is acquired by placing diodes up- and downstream of the sample to record the intensities of incoming and transmitted X-ray beams
- By placing a 2D detector behind the sample, X-ray diffraction patterns can be recorded and used to identify mineral phases within the sample (cf. X-ray diffraction below).

**Figure 2:**
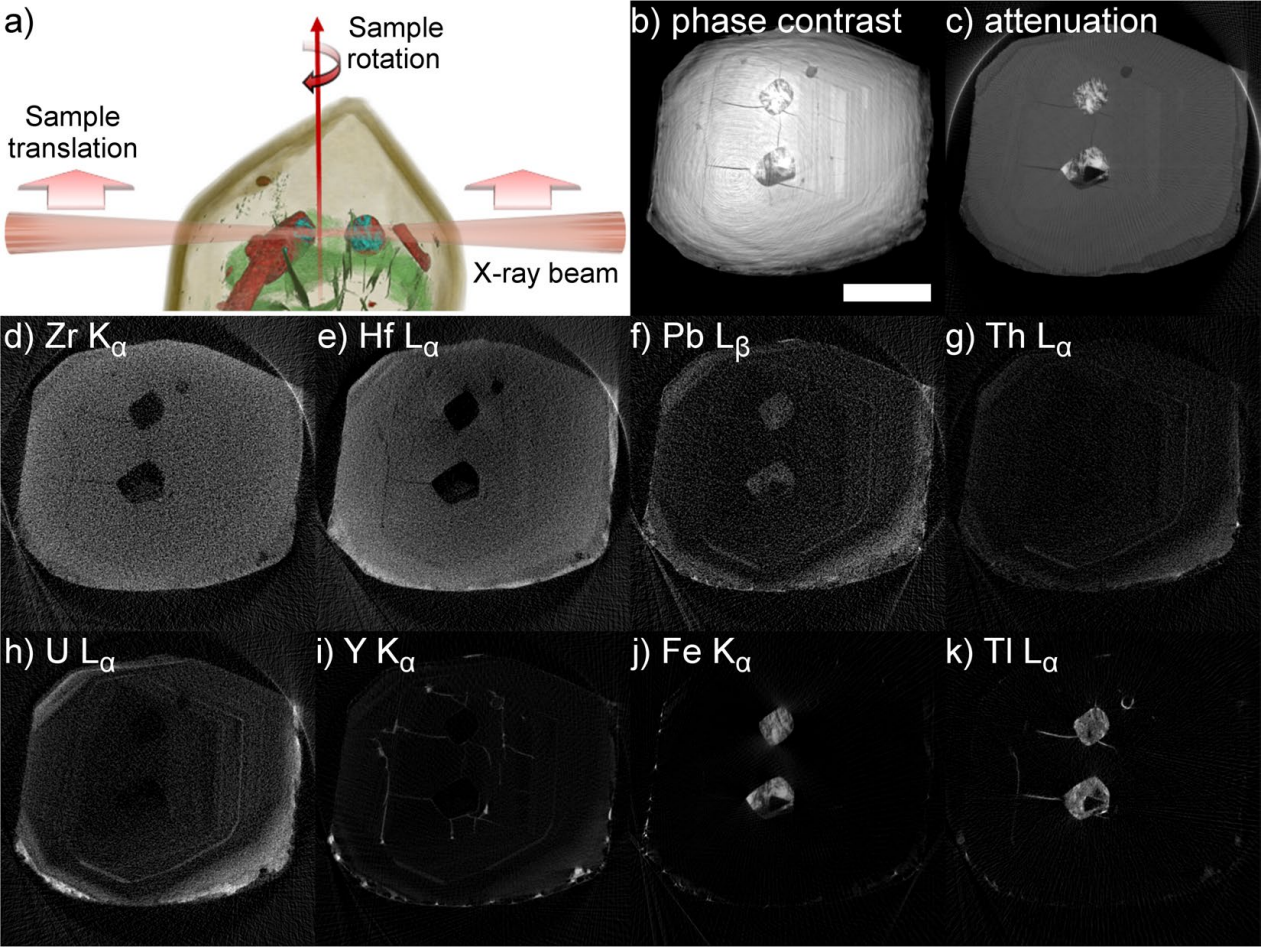
X-ray fluorescence tomography scan of a slice near the tip of zircon A1973a. (**a**) The principle of XRF tomography: the sample is placed in the focus of the X-ray beam, which illuminates one line through the sample at a time. A series of translations and rotations of the sample is then used to conduct an X-ray tomography scan in the pencil-beam geometry. Note that the divergence of the X-ray beam is strongly exaggerated for visualization purposes. (**b**) Corresponding x-y slice of the phase-contrast tomography shown in Fig. 1. Scale bar 20 µm. (**c**) Reconstruction of the absorption signal recorded from diodes placed before and after the sample. (**d–k**) Reconstructions of the intensity of characteristic X-ray emission lines of various elements, recorded with energy sensitive silicon drift detectors placed on both sides of the sample. White in the cross sectional images represents higher X-ray phase shift (**b**), absorption (**c**) or higher concentration of an element (**d**–**k**) Scale bar is 25 µm.

The principle of XRF-CT together with phase contrast and absorption reconstructions of a slice near the top of zircon A1973a are shown in Fig. 2a–c. The pure absorption contrast (Fig. 2c) reveals similar information to the phase reconstruction: most markedly the two large inclusions in the middle of the image, with an internal structure showing a bright, highly absorbing phase embedded in a matrix of lower grayvalue than the zircon. In addition, the top end of the zoning pattern, which is clearly visible in Fig. 1c–e, can be distinguished, along with some fractures conspicuously connected to the two inclusions. Less visible in the phase contrast image, but easily distinguishable in the absorption image is a less absorbing overgrowth at the edge of the zircon. Figure 2d–k show selected elemental distributions of the same cross section as revealed by XRF-CT. The zoning is most evident in the distributions of U, Pb and Th; all show a distinct oscillatory pattern in the center part of the cross section and have similar distributions, with the exception of the younger overgrowth, which is relatively depleted in Pb. The two inclusions also contain significant trace Pb. The rare earth element yttrium, on the other hand, appears concentrated in fractures, although it also shows a weak oscillatory zoning in the zircon. Of the lanthanide REEs, a slightly higher concentration of Nd, Sm, Eu and Gd can be distinguished in one fracture, and a faint oscillatory zoning similar to the Y, U, Th, and Pb distributions in Yb. It should be noted that imaging the heavy REEs Tb, Dy, Er, Tm and Lu is difficult due to overlaps of the fluorescence spectra with stronger emission lines of Fe and Hf. Major impurity element Hf exhibits zoning with higher concentration on the outer edge of the zircon, while Fe is present within the inclusions. The remaining major contribution to the fluorescence spectrum comes from Tl, which is present in inclusions and fractures.

**Figure 3:**
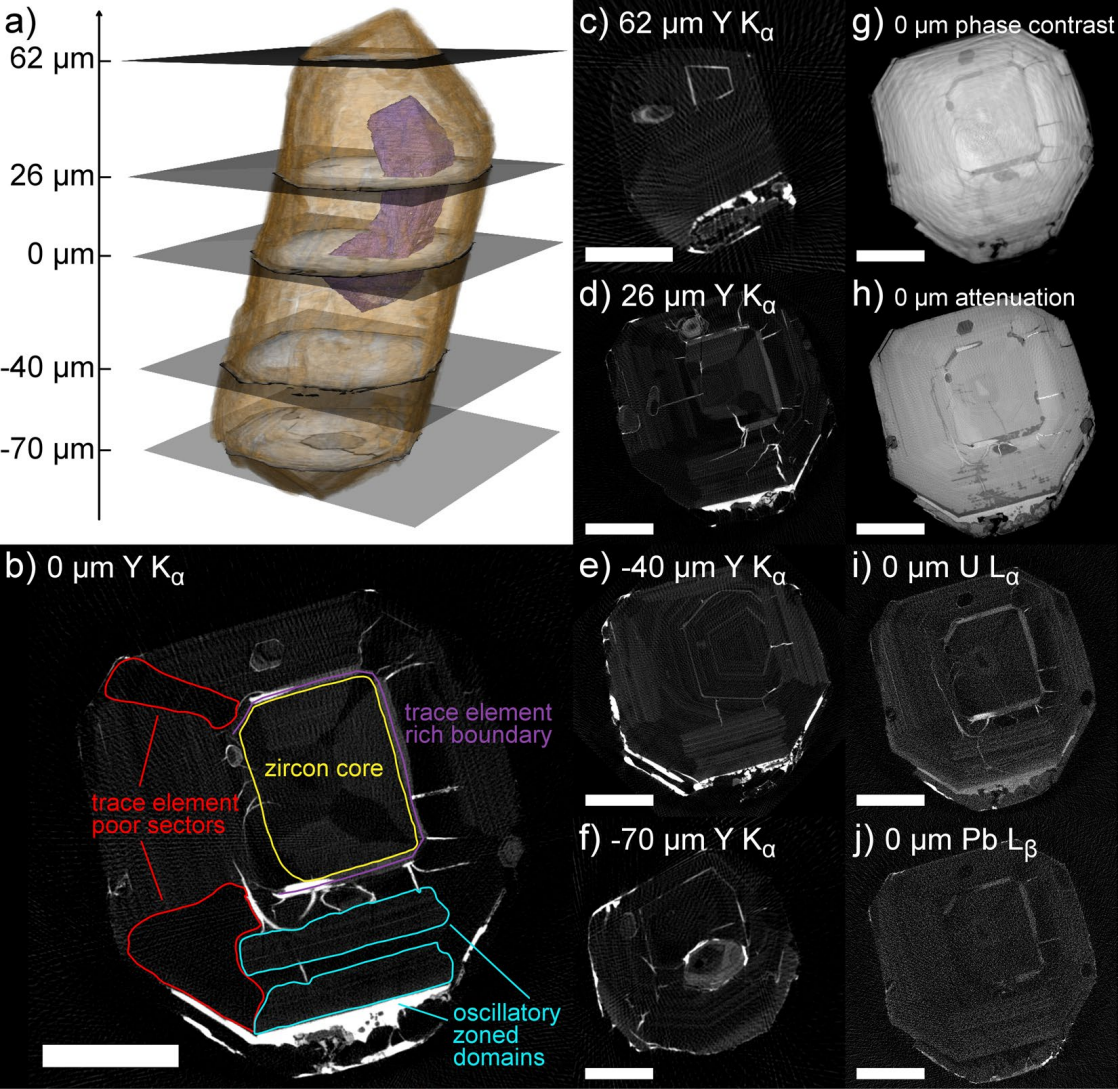
(**a**) 3D rendering of zircon 1971A2, based on the phase contrast tomography. The zircon is rendered in brown, and the dark zone found to be enriched in U, Pb, Th and Y is rendered in purple. Cross-sectional slices indicate the positions where XRF and absorption tomography was performed. (**b–f**) XRF tomographic reconstructions of Y K_α_ fluorescence line intensity at different axial cross-sections. (**g**) Phase contrast reconstruction at height 0 µm. (**h**) Absorption reconstruction at height 0 µm. (**i–j**) Reconstructions of U L_α_ and Pb L_β_ fluorescence line intensities at height 0 µm. All scale bars are 22.5 µm.

XRF-CT of zircon A1971 (Fig. 3) depicts the importance of viewing the zoning pattern at several cross-sections of the crystal. Already from the elemental maps, the isolated dark region observed in full field tomography (rendered in purple in Fig. 3a) can be seen to be enriched in Y, U, Pb, Yb and Th (Fig. 3b,d,g–j). This trace-element rich domain does not interrupt the very clear zoning pattern seen in the Y, U, Pb and Th (not shown) distributions, which is a combination of oscillatory and sector zoning: oscillatory zoned domains are interrupted by sectors with low trace element concentration. A cross-section below the end of the Y, Pb, Th and U -rich zone (Fig. 3e) reveals oscillatory zoning with a different orientation, related to the tapered end of the trace element rich zone. At the tips of the crystal (Fig. 3c,f), the Y distribution is mostly showing oscillatory zoning aligned with the faces of the crystal. Higher Y concentration is observed throughout the zircon in all inclusions and along fractures. An interesting detail are the areas of high X-ray attenuation (Fig. 3h) within the generally trace-element rich zone (which is less attenuating than the zircon) and near the edge of the crystal, separating the euhedral zircon from a non- oscillatory zoned rim: these regions have high Y and U content, but are depleted in Pb. Also the fractures are enriched in U, but not in Pb, while neither U nor Pb is detected in the inclusions. REE and Tl patterns follow roughly similar trends as in zircon A1973a: Tl is present around or in inclusions and in fractures, Yb is oscillatory zoned, while the light lanthanides (up to Gd) are mostly present along fractures and in some cases in inclusions.

**Figure 4:**
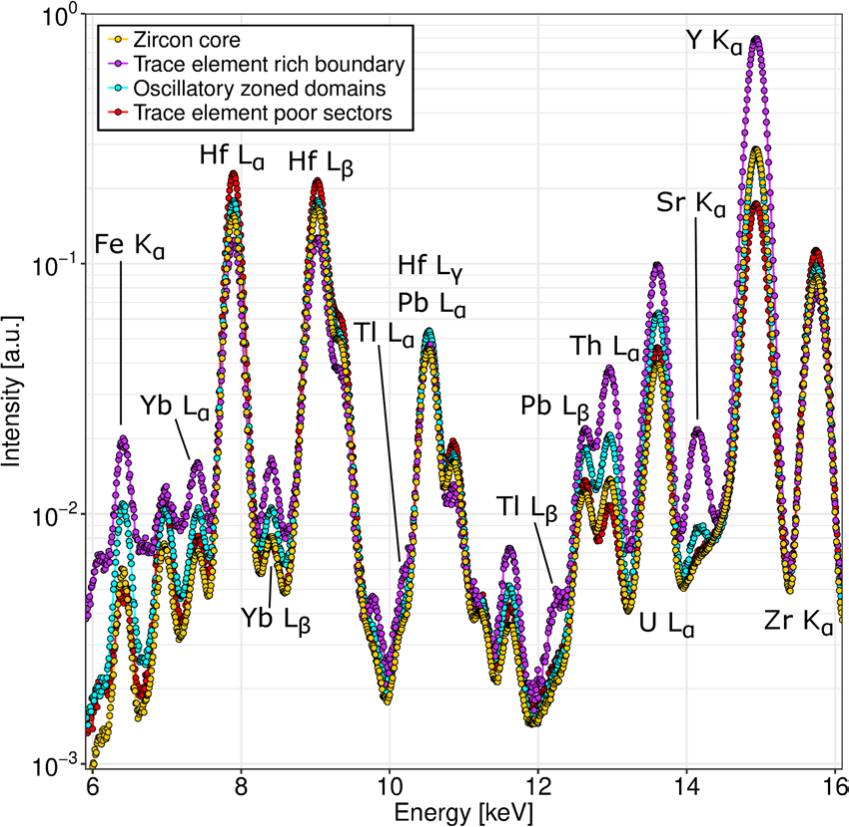
XRF spectra corresponding to the four different regions outlined in Fig. 3b. The zircon core in orange, the trace element rich zone surrounding the core in purple, non-zoned sectors outside the core in red, and oscillatory zoned domains in cyan. Major X-ray fluorescence peaks are indicated, and all spectra are normalized to the number of pixels in the corresponding region.

A major benefit of the adopted channel-wise reconstruction of XRF data (see analytical methods for details), is that from the reconstructed data, fluorescence spectra can be extracted for arbitrary subregions of the sample. Figure 4 shows such spectra for four regions in the central cross-section of zircon A1971, as indicated in Fig. 3b: the core of the zircon, the trace element rich boundary zone surrounding the core, non-zoned sectors outside of the core and oscillatory zoned domains outside the core. By fitting these spectra with the expected peak positions of elements found in the zircon, the distribution of different trace elements can be put on a more quantitative footing. This also allows to estimate the concentrations in different regions of the cross-section of the elements which are difficult to image due to overlaps with stronger fluorescence lines (mainly heavy REE other than Yb). Table 1 shows the fitted intensities of fluorescence lines for Y, U, Th, Pb, Hf and all of the REE for each of the above mentioned regions, normalized to the number of pixels in that region. In the last column, the same values are given for the entire cross-section, which allows to determine in which elements each of the regions is enriched or depleted in. Most notably, from the relative enrichment of Lu and Tm in the oscillatory zoned region (cyan in Fig. 3b), and their relative depletion in the non-zoned sectors (red in Fig. 3b), we can deduce that these elements most likely share the same oscillatory zoned distribution as Yb, even though imaging their distribution is difficult due to overlapping Fe and Hf peaks. The light REE, on the other hand, are depleted in both the oscillatory zoned and non-zoned regions, which is reflective of the fact that they are mostly found within fractures (cf. Fig. 3b: fractures are naturally included in the entire cross section, but not in the regions marked with cyan or red).

**Table 1:** Results of fitting relative intensities of different X-ray fluorescence lines to the XRF spectra shown in Fig. 4, with comparison to the spectrum over the entire cross-section.

A1971: 0 µm	Zircon core			Trace element rich boundary			Oscillatory zoned domains			Trace element poor sectors			All sample	
Fluorescence line	Normalized intensity	Fit error [%]	I/I_total_	Normalized intensity	Fit error [%]	I/I_total_	Normalized intensity	Fit error [%]	I/I_total_	Normalized intensity	Fit error [%]	I/I_total_	Normalized intensity	Fit error [%]
Y K	9.534	0.1	0.85	25.877	0.2	2.31	9.124	0.2	0.82	5.828	0.2	0.52	11.179	0.0
U L	1.351	0.4	0.76	3.406	0.5	1.91	2.164	0.4	1.21	1.574	0.5	0.88	1.786	0.1
Th L	0.397	0.8	0.88	1.209	0.9	2.67	0.624	0.8	1.38	0.282	1.4	0.62	0.453	0.3
Pb L	0.812	1.1	0.92	1.303	1.6	1.48	1.158	1.1	1.31	0.816	1.6	0.93	0.881	0.4
Hf L	10.303	0.1	0.83	7.839	0.4	0.63	11.617	0.2	0.94	14.005	0.2	1.13	12.394	0.0
Sr K	0.165	1.5	0.62	0.620	1.4	2.33	0.216	1.7	0.81	0.165	2.1	0.62	0.266	0.5
Lu L	0.198	2.3	0.91	0.308	4.1	1.41	0.262	2.7	1.20	0.199	3.4	0.91	0.218	1.0
Yb L	0.429	0.9	0.89	0.832	1.4	1.72	0.586	1.0	1.21	0.397	1.3	0.82	0.483	0.4
Tm L	0.102	4.2	0.89	0.169	7.9	1.48	0.138	5.0	1.21	0.102	6.5	0.89	0.114	2.1
Er L	N/A			0.143	13.5	1.38	0.075	12.4	0.72	0.108	8.6	1.04	0.104	3.3
Ho L	0.005	58.0	0.37	0.022	55.2	1.59	0.007	67.4	0.54	0.015	29.7	1.13	0.014	13.6
Dy L	N/A			0.162	8.7	2.24	0.015	43.9	0.21	0.008	72.6	0.11	0.072	3.2
Tb L	N/A			0.073	23.1	1.83	0.013	56.0	0.31	N/A			0.040	7.2
Gd L	0.047	4.1	0.30	0.283	3.3	1.84	0.080	4.2	0.52	0.030	11.0	0.19	0.154	0.9
Eu L	0.010	18.3	0.11	0.107	10.6	1.19	0.052	7.4	0.58	0.022	18.8	0.25	0.090	1.8
Sm L	0.009	23.2	0.12	0.118	9.5	1.64	0.024	16.1	0.33	0.018	26.0	0.25	0.072	2.1
Nd L	0.007	25.8	0.07	0.148	8.0	1.66	0.010	37.5	0.11	0.018	26.2	0.20	0.089	1.5
Pr L	0.007	17.5	0.36	0.017	33.4	0.97	0.006	34.2	0.35	0.007	44.1	0.37	0.018	4.7
Ce L	N/A			0.058	13.0	0.98	N/A			0.025	15.0	0.42	0.059	1.4
La L	N/A			0.014	45.1	1.39	0.006	40.5	0.58	0.008	42.8	0.76	0.010	7.7

The normalized intensities are the areas under the peak, curve normalized to the number of pixels in the region. The four named regions correspond to Fig. 3b, with the third subcolumn showing the normalized intensity divided by that of the whole cross-section: this indicates whether the element is enriched (>1.0) or depleted (<1.0) in the region. N/A means the fluorescence line was not reliably detected (relative error of the fit >100%).

**Figure 5:**
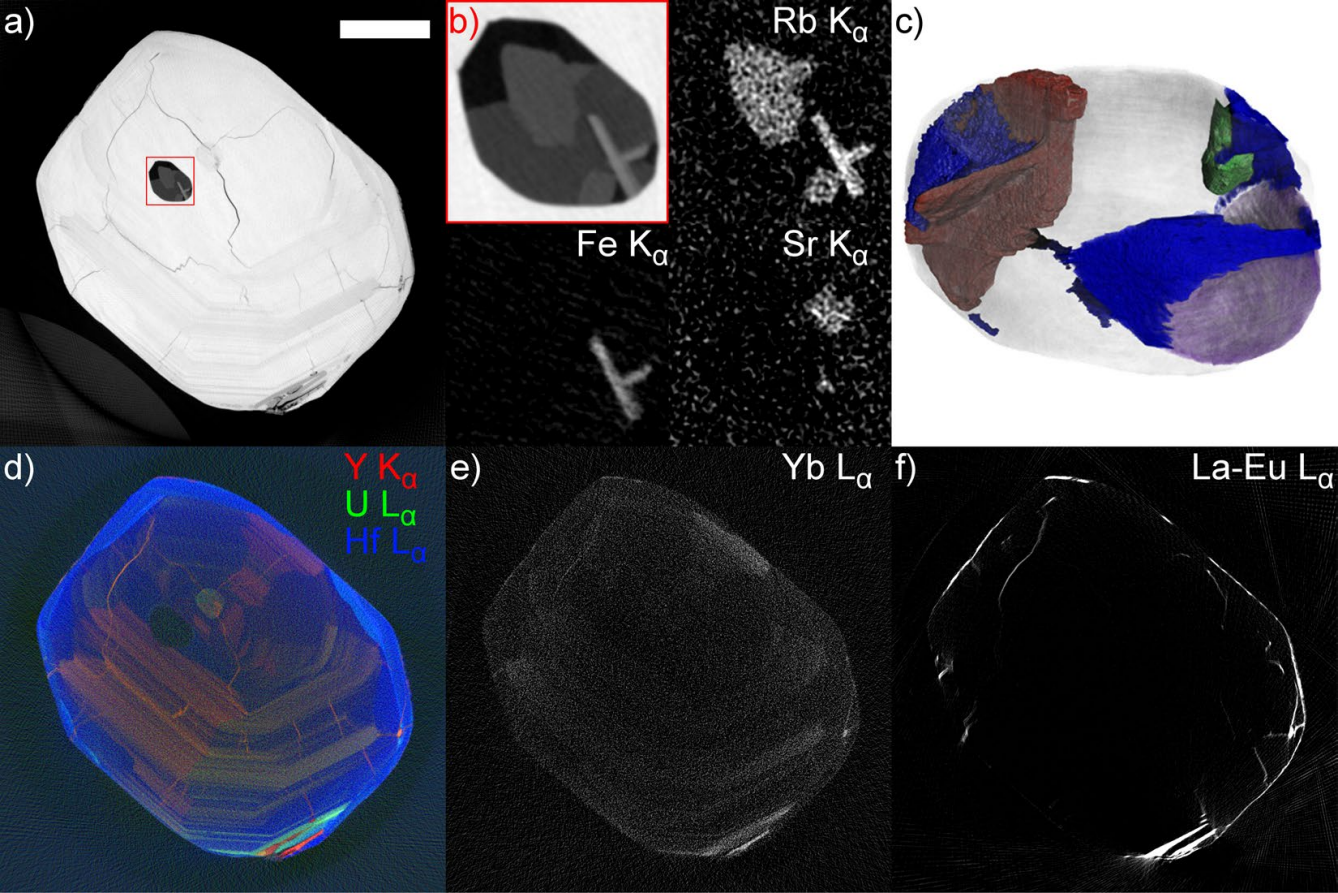
Absorption, fluorescence and phase-contrast tomography results of zircon 1973B. (**a**) Absorption reconstruction, showing concentric oscillatory zoning, cracking in the homogeneous regions of the zircon, and revealing the heterogeneous composition of a large inclusion. Scale bar is 25 µm. (**b**) Close-up of the inclusion in absorption, and intensities of Rb, Fe, and Sr K_α_ fluorescence line intensities within the inclusion. (**c**) High resolution phase-contrast tomography of the inclusion reveals 5 components with distinct X-ray refractive index. Rendered in brown: the Fe-rich crystals, which in 3D are revealed to be tabular rather than acicular in morphology. In green: a phase that is denser than remaining contents of the inclusion. In gray: a phase with intermediate grayvalue in phase contrast. In purple: a dark phase, presumably gaseous. In blue: a slightly brighter grayvalue phase, which judging from the meniscus-shaped interface with gaseous phase is at least in part liquid. (**d**) A composite image of Y, U, and Hf distribution in the XRF tomography scan, showing sectors with U alternatively enriched or depleted with respect to Y, and Hf enriched in the metamorphic rim of the zircon. (**e**) Reconstruction of Yb L_α_ fluorescence intensity, showing similar oscillatory zoned distribution as Y and U. (**f**) Reconstruction of the summed intensity over L_α_ fluorescence lines of REEs from La to Eu, showing no oscillatory or sector zoning, but the presence of these elements in fractures in the zircon.

Compared with A1971 and A1973a, sector zoning is less pronounced in zircon A1973b, which is characterized by a relatively continuous oscillatory zoning surrounding a homogeneous core of the crystal. Irregular fractures are evident in the areas that are homogeneous and bright in X-ray absorption (Fig. 5a). Some sector zoned texture can still be seen by superposing images of different elements: e.g. Fig. 5d shows a distinct interface between Y-rich and U-rich parts of the oscillatory zoning pattern. Unlike Y, U (and Pb, not shown) is also concentrated in the Hf-rich rim of the zircon (Fig. 5d). Th, on the other hand, shows the similar oscillatory pattern as Y, but is not concentrated on the rim. The fractures visible in absorption and holotomography images appear filled with U, Y, Tl and the light lanthanide REEs (Fig. 5f), whereas Yb (Fig. 5e) is weakly oscillatory zoned throughout the grain.

The large inclusion at the center of the zircon contains several phases with distinct absorption properties (Fig. 5a,b). The main contribution to the XRF spectrum of the inclusion comes from Fe, which is present in two crystals that appear prismatic in a 2D section, but are revealed to be tabular rather than prismatic in the segmented high-resolution tomography image shown in Fig. 5c. In addition to Fe, these platy crystals contain trace Rb, which is also enriched in two larger phases in the inclusion. The third easily distinguishable fluorescence from the inclusion is Sr, which can be seen in a smaller region to the side of the inclusion. It should be noted that XRF-CT is likely able to detect even trace quantities of these elements within the inclusion, and the X-ray fluorescence of all these elements is weak compared with the Fe XRF signal recorded from the inclusions in sample A1973a, indicating that even Fe may only be present in trace quantities in the minerals forming the inclusion. A more detailed discussion of detection limits and quantification of XRF-CT data is given in the analytical methods section. Part of the inclusion appears void with all methods: absorption-, fluorescence- and holotomography.

## X-ray diffraction

**Figure 6:**
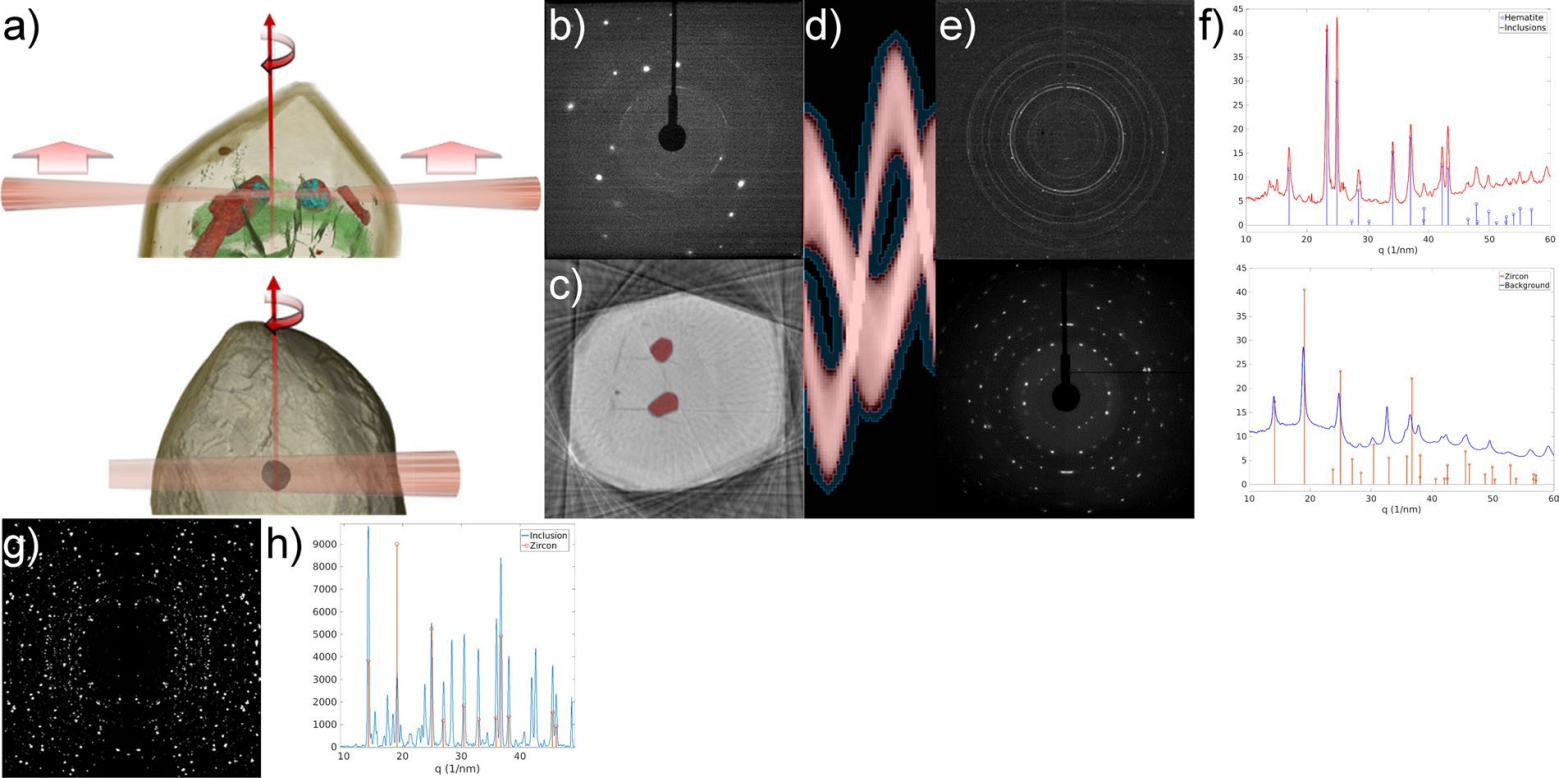
X-ray diffraction results of inclusions in zircons 1973A and 1973B. (**a**) The two acquisition modes for diffraction. On top, the pencil-beam tomographic imaging approach used with zircon 1973A: this is essentially the same as the fluorescence tomography approach (Fig. 2), with the addition of a camera behind the sample to measure one diffraction image for each translation, rotation position. Below, alternative approach used with zircon 1973B, where the inclusion is brought to the rotation axis and illuminated completely by the X-ray beam. This enables acquiring diffraction images with a much finer angular sampling needed for non-powder-like inclusions, but cannot be used to provide a diffraction tomography image of the entire sample. (**b**) Typical single X-ray diffraction image: the bright spots are diffraction peaks from the zircon, with the contribution of the inclusion only visible as faint peaks or powder ring segments. (**c**) XRF fluorescence tomography of Zr in zircon 1973A, acquired at the same time as the diffraction data. Red overlay shows the inclusions after segmentation of the image. (**d**) Forward-projected sinogram of the inclusions in (**c**). The pixels with red overlay are used for the inclusion signal in each projection, pixels with blue overlay as the ‘background’ to be subtracted. (**e**) Sum of all background-subtracted diffraction images of the inclusion (top) and of the background (bottom), which represents the zircon itself. (**f**) Azimuthally integrated patterns of (**e**), showing the expected peak positions and intensities of hematite and zircon in the stem graphs. Hematite is revealed as the primary mineral component in the inclusions, and the zircon is revealed to have a strained crystal structure with lattice parameters ~1% larger than expected from the literature. (**g**) Sum of diffraction obtained from the inclusion in 1973B, after image processing to emphasize the weaker peaks from the inclusion. (**h**) Azimuthally integrated pattern of (**g**), with the stem graph indicating peaks attributable to the zircon. The remaining peaks roughly match a plagioclase feldspar, but exact assignment of peaks remains challenging due to the presence of several crystals with unknown composition.

While zircons do not show much spatial variation in their X-ray diffraction pattern, localized X-ray diffraction can be used to non-destructively identify inclusion mineralogy. The two methods for doing this are described in Fig. 6a. The scanning approach is already described in the case of fluorescence tomography: a narrow, focused pencil X-ray beam is used to scan across the zircon, which is then rotated before the next scan. This X-ray geometry yields one diffraction pattern for each translational position at each rotation angle, and enables reconstructing the occurrence of various crystallographic phases throughout the zircon. However, due to the low number of angular steps, the phases need to be sufficiently fine-grained to produce a powder-like diffraction pattern (such as in Fig. 6e). The bottom image in Fig. 6a shows an alternative approach focusing on the mineralogical composition of one inclusion only: the inclusion is aligned with the rotation axis of the sample, and the X-ray beam size is adjusted to just illuminate the entire inclusion. The advantage is that only one diffraction image is acquired per rotation angle, which allows a much finer angular sampling. This is useful when there are only a few crystals in the inclusion or they are well aligned (i.e. the inclusion exhibits strong crystallographic texture), and only individual spots can be seen in the diffraction images.

In both cases, the major diffraction peaks in the recorded images (e.g. Fig. 6b) are those from the zircon itself; the diffraction from the inclusion is only visible as weak spots or partial rings, which require image processing techniques to be extracted and analysed. This procedure is described in detail in the analytical methods section.

Azimuthally integrating the extracted diffraction image of the inclusions in zircon A1973a shows hematite to be the major mineral component in the inclusions (Fig. 6f, top), which agrees well with the high Fe content observed in the inclusions in XRF scanning. Analysis of the background diffraction of this scan also reveals an interesting detail of the zircon crystal: comparing the integrated pattern (Fig. 6f, bottom) to the expected peak positions of zircon^32^ shows the peaks are shifted towards lower scattering angles, indicating a larger unit cell. By the positions of the (200) and (004) peaks, the lattice parameters *a* and *c* are 6.670 Å and 6.050 Å, giving approximately 1% strain respective to the literature values of 6.607 Å and 5.982 Å. A likely cause for this is metamictization: fractures leading to inclusions could also be indicative of stresses related to preferential metamictization in the zircon. Note that in this context the azimuth is the polar angle when viewing the diffraction image in the polar coordinate system, with the primary X-ray beam incident at the origin, it is not referring to geographic North.

The total integrated diffraction pattern of the polycrystalline inclusion in zircon A1973b (Fig. 6h) is more difficult to analyse, as the inclusion certainly contains several different crystalline components, each of which is only visible as individual diffraction spots, indicating that the number of crystals in the inclusion is low. The presence of several unknown phases also makes it difficult to assign individual diffraction spots to different crystals in order to refine lattice parameters. From the azimuthally integrated pattern, the major contribution from the inclusion is between the (101) and (112) peaks of zircon: the pattern matches roughly that of a plagioclase feldspar, but exactly identifying the crystal structure is difficult given the relatively weak diffraction from the inclusion.

## Discussion and conclusions

The non-destructive nature of synchrotron radiation allows 3D nanopetrography of zircons, their trace element composition, zoning and inclusions contained within them. All three zircons exhibit typical igneous zoning patterns, with well-developed oscillatory zoning cut in certain sectors by more homogeneous zones. No visible zoning is observed in the overgrowths of zircons A1971 and A1973a. XRF tomography reveals the zoning in the Y, Yb, U, Pb and Th content, where the Pb zoning is a consequence of initial U and Th zoning. The apparent zoning in the other trace elements could be caused by preferential migration of these trace elements due to radiation damage. Fitting of XRF spectra from specific regions in a cross-section suggests that the heavier REE are zoned similarly to Yb, while light REE show high concentration in fractures.

In terms of U-Pb dating, the XRF tomography reveals the distribution of the involved elements with far greater resolution than the spot sizes typically achievable with SIMS or LA-ICP-MS dating. As shown in Figs 2f–h and 3i,j, discrepancies between U and Pb distributions can be due to inclusions or cross-cutting fractures. A related phenomenon is the fractures that transect one strongly zoned domain of the crystal to another across more homogeneous-looking regions (Fig. 1). These are likely due to preferential metamictization of the U-rich zoned domains, and could provide a pathway for any mobile Pb or U leached from the zircon or present in surrounding fluid during metamorphic events.

As such, the combination of X-ray nanotomography and XRF could provide a useful tool to map locations for dating individual zircons in situations where a large population of zircons is not available. The 2D surfaces of zircons, after mounting and polishing, can be compared with the X-ray nanopetrography, as we have recently done for micrometre-scale arsenopyrite crystals^25^. Both methods are also relatively versatile in terms of scan parameters: while the high resolution XRF tomography scans presented here take several hours to acquire, a lower resolution scan with 500 nm translation steps could be acquired in approximately one hour, depending on sample dimensions. Likewise, a low-resolution 3D nanotomography scan with 400 nm voxel size could be acquired in a few minutes. A challenge for quantification of trace element concentrations remains taking into account self-absorption of the fluorescence X-rays within the sample, which will cause a decrease in intensity towards the center of the sample (cf. zircon core values in Table 1), an effect that is more pronounced for lower energy fluorescence lines. This effect also induces another trade-off to the design of an XRF tomography experiment: either the sample size or the elements available for imaging are limited by self-absorption, with the Ar K_α_ being a practical low limit for the observed fluorescence emission with the sample in air. The detection limits of XRF-CT are expected to be in the 10 ppm range for most commonly analyzed trace elements, and by scanning a suitably selected standard during the experiment should enable to interpret their spatial distribution semiquantitatively (cf. analytical methods section). In addition to allowing the extrapolation of the features observed with XRF to 3D, nanotomography also enables targeted X-ray diffraction studies of features such as inclusions within the zircon. While in this case both feldspar and hematite inclusions are already known from other samples of the same series^26^, targeted diffraction provides a complementary, non-destructive way to assess the mineralogy of inclusions within a crystal. A further benefit of diffraction is evaluation of the crystal structure of the ‘parent’ crystal: in the case of zircon A1973a the zircon crystal itself is strained, probably due to metamictization.

Overall, the unified approach presented here combining X-ray nanotomography, fluorescence and diffraction, without moving the sample from the stage, reveals a comprehensive nanoscale, 3D petrography of zircon nanostructure, providing new insight to the interplay of chemical composition and internal growth zoning, which is unattainable with methods relying on 2D images only. Moreover, comparing U, Pb, and Th distributions can help interpret results from ion beam or laser ablation techniques, while X-ray diffraction provides valuable additional information to the composition of inclusions in the crystal, which is indicative of the magmatic origins of the zircon.

The 3D nanopetrography and chemical imaging allow to compare textural relationships in the case of detrital zircon populations. Zircon is an attractive provenance proxy and different ages and geochemical signatures from a sedimentary basin or from a single sandstone sample indicate multiple sources. With the existing technology of ID16B, a relatively low-resolution (~400–500 nm) 3D nanotomography scan of 20 to 30 grains can be obtained in one day of beam time, and complemented with XRF and/or diffraction studies on representative grains during the remainder of the experiment (typically 3–6 days is reserved per experiment). These scanned grains can be mounted along with other unscanned zircons from the same sample or set of samples. For scanned grains, laser size and spot location (e.g., for ICP-MS analysis) can be placed precisely and errors in the analysis can be compared with the rest of unscanned zircons. Because of the non-destructive nature of the method, it allows the user to pinpoint locations for spot analysis on polished 2D section of zircon by matching the 3D image of individual zircons. Careful incremental polishing of the same surface can yield optimal section for spot analysis as demonstrated in our recent study^25^. We envisage that 3D textures from different age groups along with geochemical characteristics obtained from, for example LA-ICP-MS, can be compared to further constrain provenance studies.

The workflow and multimodal techniques presented in this work are by no means specific to zircon, but can be used to study the internal textures and trace element distributions of most minerals. Especially phase contrast and absorption nanotomography and diffraction studies are fairly insensitive to the exact composition of the sample, subject only to the usual trade-off between field-of-view and desired resolution common to all tomographic techniques. For X-ray fluorescence, the suitability would have to be evaluated case-by-case based on the relative absorbance of the mineral matrix to both the incoming X-ray beam and the fluorescent radiation emitted by the elements of interest. A case of particular interest could be exceedingly rare samples, where the non-destructivity of synchrotron radiation will preserve the samples for archival purposes and complementary analysis by destructive techniques such as LA-ICP-MS and electron microscopy. Examples of such rare samples could be micrometeorites or other extraterrestrial material, or e.g. Hadean zircons.

## Analytical Methods

### Sample preparation and mounting

For the X-ray analysis of individual zircons, the samples did not need any preparation apart from separating them from the host rock. For the scanning, the zircons were fixed with cyanoacrylate glue to the tip of quartz glass capillaries, which in turn were mounted on brass pins for inserting in the sample stage of the beamline. The sample mounting was identical to that described by Sayab *et al*.^25^.

### X-ray nanotomography and XRF-CT scans

The X-ray nanotomography and XRF-CT scans were performed on two experimental sessions, using an incoming X-ray energy of 17.5 keV in the ‘pink beam’ mode of beamline ID16B of the ESRF (ΔE/E ≈ 10^−2^)^23^. The beam size at focus was approximately 60 nm × 60 nm (horizontal x vertical full width at half-maximum, FWHM) for the XRF-CT scans and nanotomography of zircon A1971, and 70 nm × 60 nm for the nanotomography scans of A1973a and A1973b.

The nanotomography scans consisted of four computed tomography scans each, with 3009 projections acquired over 360°. A PCO Edge 5.5 (PCO Ag, Kelheim, Germany) camera coupled to an LSO:Tb scintillator via 10 × magnifying optics was used to record the images, using 0.4 s (A1971A) or 0.2 s (A1973a and A1973b) exposure time. The camera has 2560 × 2160 pixels and a pixel size of 6.5 µm. Sample-to-detector distances for the four scans were varied between 458.26–473.5 mm: with the camera approximately 560 mm from the X-ray beam focus, this gave an effective pixel size of 100 nm after phase retrieval. An exception to the above was the high-resolution nanotomography of the inclusion in zircon A1973b (Fig. 4), which was performed with 17.5 keV monochromatic beam (ΔE/E ≈ 10^−4^), using the PCO camera in 2 × 2 binned mode with maximum sample-to-detector distance of 550.23 mm, giving an effective pixel size of 25 nm. The CT scans of the inclusion consisted of 750 projections each, with an acquisition time per projection of 1 s.

For XRF-CT, the samples were taken to the focus of the X-ray beam, and scanned in the pencil-beam CT geometry with 100–200 nm steps and 1°–2° angular increments over a 360° rotation. These parameters varied slightly between the scans depending on the size of the sample at the cross-section to be imaged, and to avoid as much as possible refills of the ESRF storage ring (which result in some minutes of beam loss and an approximately 25% increase in beam intensity every 12 hours) occurring during the scan. The X-ray fluorescence spectra were recorded with two 3-element silicon drift detector arrays (SGX Sensortech, Corcelles-Cormondreche, Switzerland) located on either side of the sample at 90° angle to the incoming beam, and coupled to XIA-XMAP digital pulse processors (XIA LLC, Hayward, California, USA). Counting time per point was 100 ms in all scans. Photodiodes were placed upstream (before the focusing KB mirrors) and downstream of the sample to record the X-ray attenuation data.

### X-ray diffraction measurements

The X-ray diffraction was acquired with a monochromatic beam, in the transmission geometry. For the scanning diffraction tomography of sample A1973a, the X-ray energy was 25.6 keV, while 17.5 keV was used for the full-field diffraction of A1973b. The diffraction images were recorded with a fiber-optic taper version of the FReLoN F_4320T camera. Sample-to-detector distance was approximately 11 cm in both cases, and scan of lanthanum hexaboride (LaB_6_) powder was used as reference to refine the geometry.

The diffraction CT scan of A1973a was taken in the pencil-beam CT geometry, with 300 nm step size and 8.7805° angular step over 360° rotation. Acquisition dwell time (exposure + camera read-out) was 2 s per image.

For the full-field diffraction of A1973b, the entire inclusion was positioned on the rotation axis and illuminated with the X-ray beam with the help of the PCO edge imaging camera. After this, the imaging camera was replaced by the FReLoN diffraction camera, and a total of 3001 diffraction images were acquired over a 360° rotation with 1.2 s dwell time per step (0.6 s exposure + 0.6 s read-out).

### Data analysis

#### Nanotomography

After acquisition, the projection images of the four CT scans acquired of each sample were aligned and phase maps calculated using the same Paganin-like approach as Villanova *et al*.^33^, using in-house software developed at the ESRF. The phase maps were used as input to tomographic reconstruction using the ESRF PyHST software. After reconstruction, the volumetric data was semi-automatically segmented using various filtering, thresholding and volume growing tools in Avizo software (v. 9.3, ThermoFisher Scientific, Hillsboro, Oregon, USA) and 3D renderings of the data produced with VGStudioMax software (Volume Graphics GmbH, Heidelberg, Germany).

#### XRF-CT and attenuation tomography

An inhouse-developed Matlab (Mathworks Inc., Natick, Massachusetts, USA) program was used to correct the fluorescence spectra for detector dead time, normalize them to the incoming beam intensity, and perform tomographic reconstruction of both XRF and attenuation data with the filtered back-projection (FBP) method.

Typically XRF-CT data are analyzed by least-squares fitting the measured spectra to determine the contributions of characteristic X-ray emission from selected elements. This leads to one sinogram per element, which can then be tomographically reconstructed to obtain the spatial distribution of that element in the sample. In this work, an alternative approach was taken, performing tomographic reconstruction on each channel (corresponding to 10 eV) of the fluorescence spectrum individually. This produces as a result a ‘spectral image’ of the cross-section through the zircon, where each pixel has not one grayscale or color value, but an entire fluorescence spectrum associated with it. Compared with the traditional approach, this method has two main advantages:

- It enables easy comparison of XRF spectra corresponding to arbitrarily chosen regions of the sample. For example, Fig. 4 shows the comparison of fluorescence spectra corresponding to the different regions of sample A1971 outlined in Fig. 3b. Relative abundances of various elements can be determined by fitting the spectra at this stage, as shown in Table 1.
- By not fitting the spectra prior to reconstruction, it is not necessary to choose which elements of the periodic table to include in the analysis; all energies are reconstructed from the raw data, and further analysis of the reconstructed image can reveal contributions from elements not visible in the sum spectrum of the entire sample. This is the case for the Rb and Sr observed in the inclusion in A1973b (Fig. 5), in the sum spectrum especially the Rb is completely masked out by the U L_α_ fluorescence line, and only revealed through isolation of the fluorescence originating from the inclusion.

In this work, the ROI imaging toolbox of the PyMCA software^34^ was used for analysis and visualization of the spectra after reconstruction. The energy scale was calibrated using the Ar K_α_, Hf L_α_ and Y K_α_ fluorescence lines present in the sum spectrum of each sample. For the fits presented in Table 1 and Supplementary Figure S1, the MCA Advanced fit tool of PyMCA was used: the background was first removed using the ‘strip background’ method with 25 channel peak width and 4000 iterations. After this, the remaining spectrum was fitted with Gaussian peaks corresponding to the K-fluorescence lines of Ar, Ca, Ti, Cr, Mn, Fe, Kr, Rb, Sr, Y, Zr and Mo, and the L-fluorescence lines of the lanthanides (except Pm), Hf, Tl, Pb, Rn, Th and U, using Poisson weighting of the data and 30 fit iterations. The fitting region was limited to the energy range 2.5–17.25 keV.

#### XRF-CT detection limits and quantification

To estimate the detection limits reachable via XRF tomography, a fragment of the zircon 91500 standard was scanned using the same XRF tomography protocol in a later experimental session. The sum spectrum of this scan is given in the Supplementary Information, Figure S1, along with the result of fitting with the fluorescence lines of the elements discussed here. Focusing on the region between ~5–10 keV, we can attribute distinct peaks at 7.42 and 8.41 keV to Yb, and fit significant Lu, Tm, and Er, but none of the lighter REE. An exception is La, which is a known impurity in the spectra, being present in the silicon drift detectors themselves. Comparing this with the LA-ICP-MS reference values for the zircon 91500 by Wiedenbeck *et al*.^35^, which are 24.6 ppm, 6.89 ppm, 73.9 ppm and 13.1 ppm for Er, Tm, Yb, and Lu, respectively, we would expect to be able to detect these elements from the 10 ppm range. The yttrium K_α_ peak is very pronounced in all measured spectra, due to the K absorption edge (17.038 keV) being only slightly below the incoming X-ray beam energy (17.5 keV), which increases photoelectric absorption and hence also fluorescence emission by yttrium. Coupled with higher penetration of the Y K_α_ radiation in zircon, detection limits for yttrium can be expected to be significantly better than the 140 ppm quoted by Wiedenbeck *et al*. for zircon 91500. Concentrations for the REE up to Tb are significantly lower in zircon 91500, and their contributions are not discernible from the reference spectrum. However, in all but the most mafic zircon, the REE concentrations can be expected to be higher than in 91500^36^, and indeed they are detectable in the samples measured in this study, which are from granitic rock. For the heavier elements Pb, Th and U, distinct peaks are observed in zircon 91500, which puts their detection limits also in the 10 ppm range, or below, when compared to their respective LA-ICP-MS working values of 17.85, 29.9, and 80.0 ppm. Based on previous work on the same zircon populations (see appendix B to Lahtinen *et al*.^26^), the U, Th and Pb concentrations for the zircons studied in this work are higher, on average Lahtinen *et al*. measured 270 ppm, 107 ppm and 113 ppm, respectively, for sample A1973, and 661 ppm, 232 ppm and 275 ppm, respectively, for A1971. This is easily seen in Fig. 4 as much more pronounced U L_α_, Th, L_α_ and Pb L_β_ peaks.

A final thing to be noted in Fig. 4 is the intensity of the Zr K_α_ peak at 15.8 keV: zircon is roughly 50% Zr by mass, but the Zr K_α_ peak is less intense than the Y K_α_ peak, even though the Y concentration is many orders of magnitude lower. This is due to the fact that the chosen incoming X-ray beam energy of 17.5 keV is very close to the energy of the Y K absorption edge (17.04 keV), but not sufficient to ionize the Zr K electrons with a binding energy of ~18 keV; the Zr K edge is instead ionized by a higher harmonic energy of the undulator, that passes through the beamline optics. While insignificant in intensity compared to the primary beam, this higher harmonic still produces measurable Zr fluorescence because Zr is in turn many orders of magnitude more abundant in zircon than the remaining elements discussed here. This effect also provides a potential method to estimate trace element concentrations on an absolute scale: by assuming the Zr content in zircon to be the same in both samples, the peak intensities of all other elements can be normalized to the Zr K_α_ peak and compared to a known standard.

#### X-ray diffraction

Due to the varying data acquisition strategies, different analysis methods were applied to the X-ray diffraction data from the inclusions in A1973a and A1973b. In the case of the scanning approach used with A1973a, the data analysis can be divided into six steps:

1. Calculating the total diffracted intensity in the images and reconstructing with the FBP algorithm. The result is a 2D image in which the inclusions are seen as regions darker than the zircon. Alternatively, Zr fluorescence recorded at the same time can be used (Fig. 6c).
2. Segmenting the inclusions in the reconstruction, and forward-projecting the segmentation. Step 2 results in a sinogram (Fig. 6d), in which each pixel is corresponds to one diffraction image taken during the scan.
3. For each rotation angle, calculating the mean of diffraction images containing the inclusion, i.e. non-zero pixels in the sinogram, and the mean of the ‘background’, i.e. adjacent pixels in the same column, which represents the diffraction corresponding to the zircon itself. Step 3 results in two stacks of 41 diffraction images, one for the inclusions and one for the zircon background.
4. For each pair, locating the peaks present in the background by a combination of a top-hat transform and thresholding, and masking them out in the inclusion images. This step only eliminates peaks in the inclusion images.
5. Taking the maximum value for each pixel in both stacks, resulting in one image representing the accumulated diffraction from the inclusions, and another image representing the accumulated diffraction from the zircon (Fig. 6e).
6. Calibration of the experimental geometry, dark image subtraction, and azimuthal integration of the accumulated images, giving 1D powder diffraction patterns (Fig. 6f).

The full-field diffraction scan used in the case of the inclusion in A1973b is conceptually simpler: diffraction from both the inclusion and zircon is present in every image. The only image processing used in this case was to apply a top-hat transform (commonly used to isolate small bright objects in an image) to the entire stack of 3001 images, in order to emphasize the contribution of the inclusion, which would otherwise be very weak compared to the zircon peaks. After this, the accumulated diffraction pattern was calculated and azimuthally integrated as in steps 5–6 above.

In this work, in-house developed Matlab scripts were used for the FBP reconstruction, calculating the top-hat transforms and thresholding and segmentation of images (steps 1–5 above), PyHST was used for the forward projection (part of step 2 above), and the XRDUA software^37^ for calibrating the acquisition geometry, background subtraction and azimuthal integration of the accumulated images (step 6).

### Data Availability

The datasets generated during and/or analysed during the current study are available from the corresponding author on reasonable request.

## Electronic supplementary material

Supplementary figure S1

Supplementary video 1
