# Supplementary material for: 3D nanopetrography and chemical imaging of datable zircons by synchrotron multimodal X-ray tomography
Source: Sci Rep. 2018 Mar 16;8:4747. doi: 10.1038/s41598-018-22891-9 (PMC5856831; doi:10.1038/s41598-018-22891-9)
Supplement: Supplementary file 1 — Supplementary figure S1 [file 41598_2018_22891_MOESM1_ESM.docx]

3D nanopetrography and chemical imaging of datable zircons by synchrotron multimodal X-ray tomography

**J.-P. Suuronen^1^ and M. Sayab^2^**

*^1^ESRF – The European Synchrotron, CS40220, 38043 Grenoble Cedex 9, France*

*^2^Geological Survey of Finland, P.O. Box 96, FI-02151 Espoo, Finland*

**Supplementary material:**

**
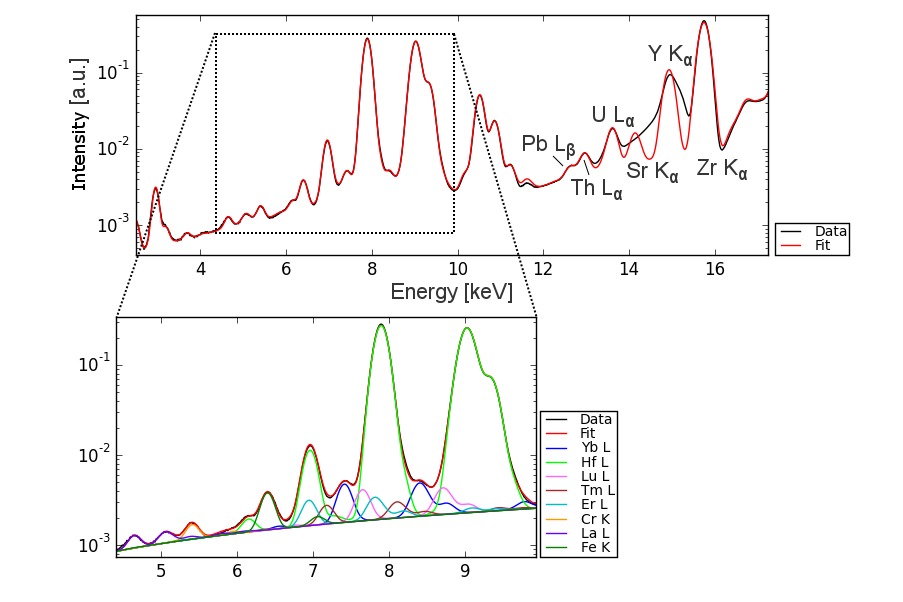
**

**Supplementary figure S1.** Normalized X-ray fluorescence spectrum (black) of a fragment of the zircon 91500, along with fit with the expected fluorescence peaks of Hf, U, Th, Pb, Sr, the REE, Fe, and some additional elements known to be present on the beamline. The inset shows the contributions of the heavy REEs Yb, Lu, TM, and Er in the vicinity of the Hf L fluorescence lines.
